# Thermo-L-Asparaginases: From the Role in the Viability of Thermophiles and Hyperthermophiles at High Temperatures to a Molecular Understanding of Their Thermoactivity and Thermostability

**DOI:** 10.3390/ijms24032674

**Published:** 2023-01-31

**Authors:** Maria Dumina, Alexander Zhgun

**Affiliations:** Group of Fungal Genetic Engineering, Federal Research Center “Fundamentals of Biotechnology of the Russian Academy of Sciences”, 117312 Moscow, Russia

**Keywords:** thermophiles, L-asparaginase, thermo-ASNase, key metabolic enzyme, thermostability, structural features

## Abstract

L-asparaginase (L-ASNase) is a vital enzyme with a broad range of applications in medicine, food industry, and diagnostics. Among various organisms expressing L-ASNases, thermophiles and hyperthermophiles produce enzymes with superior performances—stable and heat resistant thermo-ASNases. This review is an attempt to take a broader view on the thermo-ASNases. Here we discuss the position of thermo-ASNases in the large family of L-ASNases, their role in the heat-tolerance cellular system of thermophiles and hyperthermophiles, and molecular aspects of their thermoactivity and thermostability. Different types of thermo-ASNases exhibit specific L-asparaginase activity and additional secondary activities. All products of these enzymatic reactions are associated with diverse metabolic pathways and are important for mitigating heat stress. Thermo-ASNases are quite distinct from typical mesophilic L-ASNases based on structural properties, kinetic and activity profiles. Here we attempt to summarize the current understanding of the molecular mechanisms of thermo-ASNases’ thermoactivity and thermostability, from amino acid composition to structural–functional relationships. Research of these enzymes has fundamental and biotechnological significance. Thermo-ASNases and their improved variants, cloned and expressed in mesophilic hosts, can form a large pool of enzymes with valuable characteristics for biotechnological application.

## 1. Introduction

Thermophiles and hyperthermophiles are unique microorganisms that not only survive but also thrive at high temperatures. Among environmental factors temperature is unique in that it crosses physical barriers [1]. As a result, organisms cannot efficiently shield themselves from temperature in the way they can shield themselves from extreme external pH or salinity by maintaining steep concentration gradients over biological membranes. Each biomolecule inside microorganisms must be adapted to the temperatures in which they grow [1].

Thermophiles and hyperthermophiles are generally categorized based on the optimum growth temperatures. No consensus has been reached regarding the exact temperature range of each category [1,2,3]; in this review we use the following: thermophiles (TP) grow in the range of temperatures from 50 °C to 80 °C, whereas hyperthermophiles (HP) live in extremely hot environments with temperatures over 80 °C.

To thrive in such habitats, microorganisms use a combination of adaptive mechanisms. Studies of these specific mechanisms are carried out on each level of hierarchy, from microbial communities to cells and molecules. The area of special interest is elucidation of the molecular basis of thermophilic proteins’ stability and functionality under extreme conditions. Intrinsic resistance of proteins to high temperatures remains a fascinating puzzle of fundamental significance. Moreover, understanding the mechanisms underlying their thermostability is key for designing enzymes that can work in harsh conditions. Thus, studying the structural and functional features of the thermophilic/hyperthermophilic proteins as important basic components of metabolic pathways creates the basis to obtain highly active and stable forms of biotechnology relevant enzymes with broad prospects.

Among the most important industrial enzymes, L-asparaginase (L-ASNase) stands out. L-ASNase (EC 3.5.1.1; L-asparagine amidohydrolase) catalyzes the conversion of the amino acid L-asparagine (L-Asn) to L-aspartic acid and ammonia [4] and is used in the pharmaceutical, biosensor and food industries [5,6,7].

The enzyme is firstly known as an important anticancer agent used for treatment of acute lymphoblastic leukemia and other related blood cancers worldwide. The enzyme selectively hydrolyzes the extracellular L-Asn. The inability of susceptible tumor cells to synthesize their own L-Asn leads to death of lymphoblastic cells by apoptosis [8,9].

L-ASNase is used in the food industry to prevent the acrylamide formation in commercial fried foods [10]. By catalyzing the hydrolysis of L-Asn, L-ASNase prevents the reaction of reducing sugars with this amino acid to form carcinogenic acrylamide [11].

L-ASNase is applied in biosensors for monitoring the level of L-Asn both for diagnostic purposes and in the food industry [12,13].

Low stability of the current commercial L-ASNases from mesophiles restricts enzyme industrial application, particularly in the food industry, where temperatures shoot up to 120 °C or even beyond, resulting in a relatively rapid loss of their activity.

Relatively recently, thermophiles, and in particular hyperthermophiles, have been reported for their potential to produce L-ASNases with extraordinary properties. L-ASNases have been already characterized from archaea *Thermococcus kodakaraensis* [14,15,16], *Thermococcus zilligii* [17], *Thermococcus gammatolerans* [18], *Thermococcus sibiricus* [19], *Pyrococcus yayanosii* CH1 [20], *Pyrococcus furiosus* [21,22,23,24], *Pyrococcus horikoshii* [25], *Pyrococcus abyssi* [26], *Archaeoglobus fulgidus* [27]; *Pyrobaculum calidifontis* [28], living under extremely high temperatures. Due to superior performances for different areas of biotechnology [29], investigation of the characteristic features of thermophilic/hyperthermophilic L-ASNases (thermo-ASNases) is of particular interest.

On the other hand, these enzymes carry out a set of specific essential tasks in the thermophilic/hyperthermophilic cells.

In this review, we discuss the role of thermo-ASNases in the viability of their host organisms at high temperatures. Summarizing data, a complex action of these enzymes, important for survival under high temperature stress, is proposed. These results are integrated with the established structural basis of thermostability of thermo-ASNases. Thus, the review represents a complex assessment of cellular and molecular aspects of high-temperature tolerance of thermophiles based on L-ASNases as key metabolic enzymes. In turn, it summarizes the findings contributing to the ways for further rational investigation of thermo-ASNases perspective for biotechnological application.

## 2. Thermophilic L-ASNases as Members of a Large Family: Types Identified and Classification Issues

L-ASNases are an amazingly diverse family of enzymes [30]. These enzymes have been isolated from various organisms including bacteria, archaea, fungi, and eukarya [6,31,32,33].

According to the historical classification, L-ASNases are divided into three major groups depending on amino acid sequence, expressing organism, substrate specificity, structural and biophysical properties: bacterial-type I and II L-asparaginases, plant-type L-asparaginases, and Rhizobial-type L-asparaginases [31]. Despite widespread acceptance, this classification, based on the source of the first enzymes discovered, can be misleading. L-ASNases of the three canonical types, i.e., bacterial-type, plant-type and *R. etli*-type, are found throughout several kingdoms of life [34,35,36,37].

The preponderance of L-ASNases studied are found to be optimally active at, or near, mesophilic temperatures (approximately 30–40 °C) and belong to bacterial-type L-asparaginases [38]. Thermophilic/hyperthermophilic counterparts are not so extensively characterized. According to the classification originally used for mesophilic enzymes, three types of thermo-ASNases are currently reported: bacterial-type I, bacterial-type II, and plant-type L-asparaginases.

Cytosolic bacterial type I L-ASNases are involved in nitrogen metabolism and appear to be expressed constitutively [30,39]. In mesophiles, these enzymes exhibit low affinity towards L-Asn with K_M_ values in the millimolar range [35].

Periplasmic bacterial type II L-ASNases appear to participate in carbon metabolism and their expression is tightly regulated by different factors [39]. Type II enzymes of mesophilic origin exhibit high specific activity towards L-Asn with micromolar K_M_.

Plant-type L-ASNases have dual L-asparaginase and isoaspartyl aminopeptidase activity [35]. These enzymes are located in the periplasmic space and hydrolyze the side-chain amide bond of L-Asn or its β-peptides [40].They are low-affinity proteins (millimolar K_M_) that belong to the superfamily of N-terminal nucleophile hydrolases [35,40,41].

**Figure 1:**
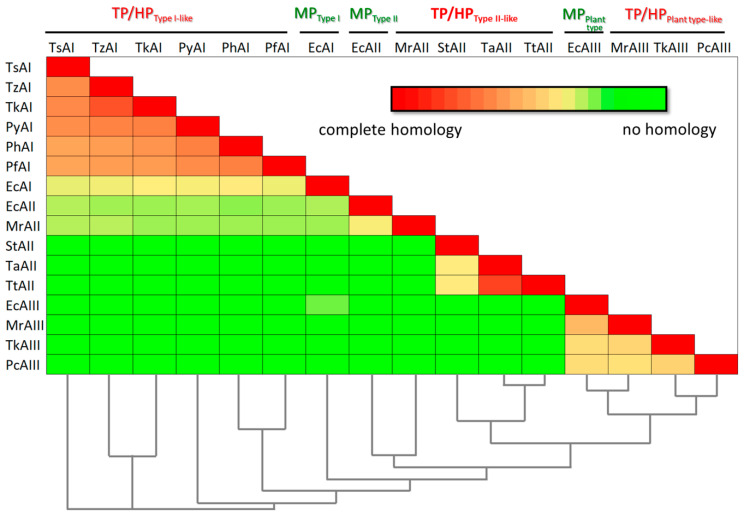
Amino acid identity matrix and phylogenetic relationships of mesophilic (MP), thermophilic (TP), and hyperthermophilic (HP) L-asparaginases from: TsAI—*Thermococcus sibiricus* (Accession Number: WP_015849943.1); TzAI—*Thermococcus zilligii* (Accession Number: WP_010478656.1); TkAI—*Thermococcus kodakarensis* (Accession Number: WP_011250607.1); PyAI—*Pyrococcus yayanosii* (Accession Number: WP_013906452.1); PhAI—*Pyrococcus horikoshii* (Accession Number: WP_010884185.1); PfAI—*Pyrococcus furiosus* (Accession Number: WP_011013191.1); EcAI—*Escherichia coli* (Accession Number: NP_416281.1); EcAII—*Escherichia coli* (Accession Number: AAA23445.1); MrAII—*Melioribacter roseus* (Accession Number: WP_014855710.1); StAII—*Streptomyces thermoluteus* (Accession Number: BAJ25701.1); TaAII—*Thermus aquaticus* (Accession Number: WP_003047338.1); TtAII—*Thermus thermophilus* (Accession Number: BDB12174.1); EcAIII—*Escherichia coli* (Accession Number: P37595.2); MrAIII—*Melioribacter roseus* (Accession Number: WP_014855981.1); TkAIII—*Thermococcus kodakarensis* (Accession Number: Q5JHT1.1); PcAIII—*Pyrobaculum calidifonti* (Accession Number: ABO08395.1).

According to the classification commonly used, most of the characterized enzymes from hyperthermophilic archaea *Pyrococcus* sp. and *Thermococcus* sp. belong to bacterial-type I L-ASNases (Table 1) [14,19,25]. The degree of identity of L-ASNases of the *Thermococcus* genera is about 60–80%, and for L-ASNases of *Pyrococcus* it is slightly lower (54–65%) (Figure 1) [19,33].

At the same time, the sequence identity between hyperthermophilic bacterial-type I L-ASNases from archaea and type I L-ASNase from mesophilic bacteria *Escherichia coli* (EcAI, GenBank accession no. NP_416281.1, encoded by the *ansA* gene) used as a representative of this type [30,38] does not exceed 37% (Figure 1).

Most of the identified enzymes from thermophilic bacteria represent bacterial-type II L-ASNases (Table 1). Examples include enzymes from *Streptomyces thermoluteus* subsp. fuscus NBRC 14270 (GenBank accession no. BAJ25701.1) [46], *Melioribacter roseus* (GenBank accession no. WP_014855710.1) [49], *Thermus thermophilus* (GenBank accession no. BDB12174.1), *Thermus aquaticus* (GenBank accession no. WP_003047338). Although being defined as type II L-ASNases, poor sequence identity between some of these enzymes is revealed: while the degree of identity between L-ASNases from *Thermus* sp. is more than 80%, the enzyme from *Streptomyces thermoluteus* subsp. fuscus NBRC shares only ~37% identity with L-ASNases from *Thermus* sp. No obvious sequence similarity (identity 10–12%) is found between these enzymes and L-ASNase from *M. roseus*. According to amino acid sequence alignment, type II L-ASNase from *E. coli* (EcAII, ansB, GenBank accession no. AAA23445.1), being a typical example of bacterial-type II ASNases, displays only 35.2% identity with *M. roseus* L-ASNase and 11–12% with counterparts from thermophilic bacteria mentioned above (Figure 1).

To date, the first three plant-type thermo-ASNases have been reported: from archaea *Pyrobaculum calidifontis* (ABO08395.1) and *Thermococcus kodakarensis* (Q5JHT1.1), and bacteria *M. roseus* (WP_014855981.1) [28,43,49]. Sequence identity in this group of thermo-ASNases does not exceed 39.5–43.8%. When compared with mesophilic plant-type L-ASNase from *E. coli* (EcAIII, ybiK, P37595), a similar degree of identity is observed—40.7–50.5% (Figure 1).

Although characterization of thermo-ASNases is far from complete, large variations in their properties are already evident, this being in keeping with the poor sequence identity between L-ASNases of TP/HP and mesophilic origin, as well as within the thermo-ASNase group.

In general, an increasing need for a revision of L-ASNases classification is noted in recent reviews [38,51]. While Schultz da Silva et al. have proposed to rename bacterial-type L-asparaginases, plant-type L-asparaginases, and Rhizobial-type L-asparaginases as Class 1, Class 2 and Class 3, respectively [51], Lubkowski and Wlodawer are the first who suggested separating HP archaeal L-ASNases in a distinct subgroup [38]. The authors proposed to group L-ASNases into three categories: tetrameric enzymes of type I and type II, and dimeric archaeal enzymes (HP L-ASNases) [38].

Unlike tetrameric mesophilic enzymes, L-ASNases expressed in HP archaea are homodimeric and sufficiently divergent. The enzymes display structural and topological properties that are distinct from either typical type I or type II L-ASNases, have active sites that deviate from the canonical arrangement seen in type I or II ASNases, or display kinetic/activity profiles that are at the intersection between type I and II ASNases [38].

Agreeing with the opinion of Lubkowski and Wlodawer, it should be noted that only the first characterized archaeal L-ASNases—*Pyrococcus horikoshii* (PhA), *Pyrococcus furiosus* (PfA), and *Thermococcus kodakarensis* (TkA)—were taken into consideration [38]. According to recent data, TP/HP microorganisms can possess several enzymes of different types exhibiting L-asparaginase activity [43,49]. For example, hyperthermophilic archaeon *T. kodakarensis* contains not only TkA more similar to members of the type I [14], but also a newly discovered TK2246 classified as a plant-type L-ASNase [43]. TK2246 displays low similarity with both TkA and typical plant-type L-ASNase *E. coli* EcAIII. In the pioneering research, homology modelling revealed structural changes between TK2246 and EcAIII—the presence of additional loop in TK2246 compared to EcAIII [43]. Moreover, gel filtration chromatography and SDS-PAGE indicated that TK2246 exists in a homodimeric form comprising two identical subunits [43]. At the same time, known mesophilic plant-type L-ASNases, including EcAIII, are heterotetramers or dimers of two αβ heterodimers [30,34]. In this view, it is likely that the subgroup of archaeal L-ASNases should be further divided into additional types.

The proposed classification of Lubkowski and Wlodawer concerns only the consideration of thermo-ASNases from archaea as a separate subgroup; nevertheless, thermo-ASNases of bacterial origin are also quite distinct from typical mesophilic L-ASNases. Also, there is no structural evidence, but it is supposed, that bacterial thermo-ASNases can act in oligomeric state different from di- or tetrameric, for example hexameric L-ASNase *Thermus thermophilus* (trimer of dimers) [30,47].

Initial classification criteria, such as comparison of amino acid homologies to EcAI and EcAII and/or substrate affinities (millimolar vs. micromolar, expressed in terms of K_M_) are also hardly applicable to thermophilic L-ASNases. For example, micromolar affinity to L-asparagine is defined as a specific trait of bacterial type II L-ASNases. Nevertheless, known type II L-ASNases from thermophilic bacteria show K_M_ values in the millimolar range [6,49]. The level of substrate affinity in thermo-ASNases predetermined not by type, but rather the fact of adaptation of these organisms to high temperatures, far exceeding the appropriate for mesophiles. Increased K_M_ values of various enzymes originating from thermophilic microorganisms compared to mesophilic counterparts confirm this conclusion: phosphoglycerate kinase [52], glutamate dehydrogenase [53], alkaline phosphatase [54,55,56], GTPase (TrmE) [57] and glucose-6-phosphate dehydrogenase [58]. In fact, successful attempts to improve the thermal stability of mesophilic L-ASNases have also resulted in a concomitant increase in K_M_ [59]. It appears that adaptation at high environmental temperatures involves an increase in K_M_ and k_kat_ for thermo-ASNases [19]. This characteristic feature of thermo-ASNases allows optimizing catalytic efficiency by reaching a balance between substrate binding and the rate of product release.

**Figure 2:**
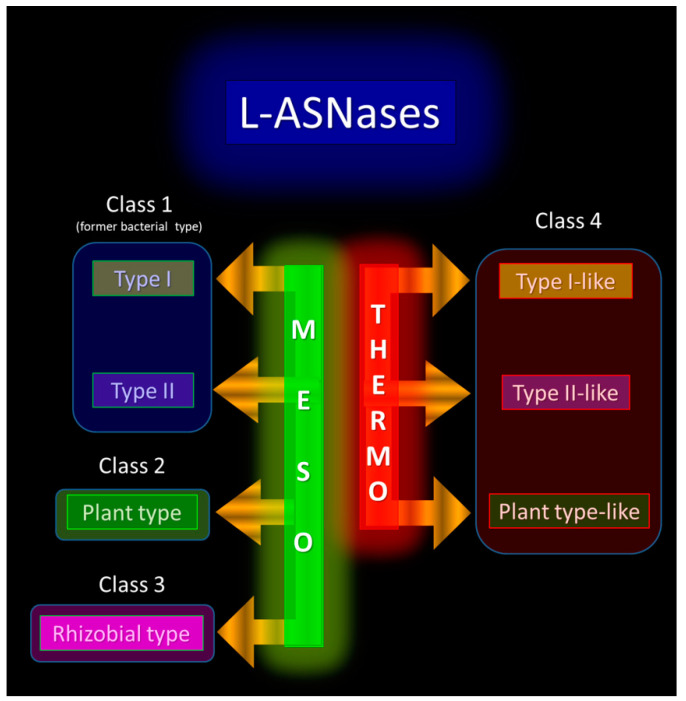
Proposed classification of L-ASNases, including thermo-ASNases as a novel Class 4.

In summary, there is a clear need to revise the current classification of L-ASNases. Considering the striking differences between mesophilic and TP/HP enzymes, probably associated with differences in the living environments, it must be recognized that thermo-ASNases must form a unique and novel group in the large family of L-ASNases and must be appropriately subdivided inside the group. This may be an extension of the classification proposed by Schultz da Silva et al., where thermo-ASNases would be classified into Class 4. Based on similarity in tertiary structures and general mechanisms within previously accepted types, and in an attempt to distinguish thermo-ASNases from mesophilic ones, Class 4 can be further subdivided into type I-like, type II-like, and plant-type-like (Figure 2).

## 3. L-ASNases as Key Metabolic Enzymes for Thermophilic and Hyperthermophilic Microorganisms

**Figure 3:**
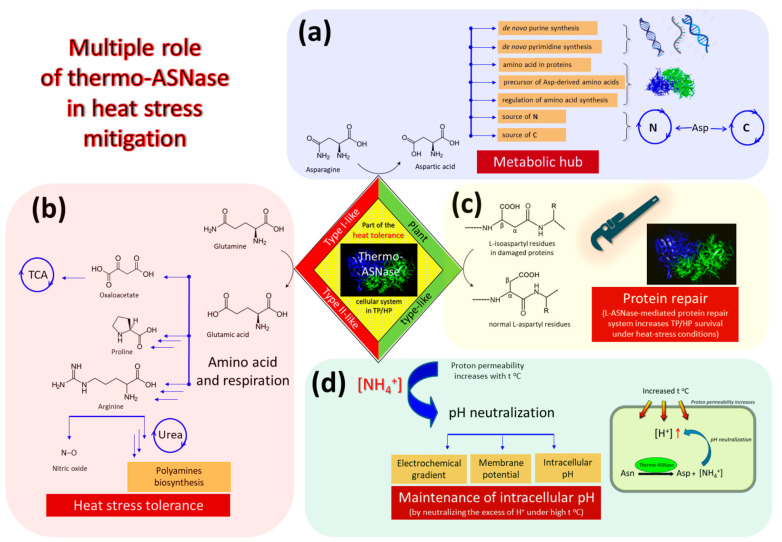
Proposed scheme of multiple effects of thermo-ASNases on the survival of thermophilic (TP) and hyperthermophilic (HP) microorganisms under heat stress. Asp-derived—aspartate-derived, N—nitrogen, C—carbon, TCA—tricarboxylic acid cycle. (**a**) Aspartic acid, formed from asparagine by the action of thermo-ASNases, is associated with multiple metabolic pathways. (**b**) The role of thermo-ASNases in heat stress tolerance associated with the conversion of glutamine to glutamic acid: intensification of respiration and metabolism of amino acids. (**c**) Isoaspartyl aminopeptidase activity of thermo-ASNases as part of the protein repair system under heat-stress. (**d**) Maintenance of intracellular pH mediated by thermo-ASNases: thermo-ASNases by releasing ammonium ions can neutralize the excess proton influx under high temperatures. The diamond borders represent type I-like, type II-like and plant type-like thermo-ASNases. The red border is type I-like and type II-like thermo-ASNases associated with functions (**a**,**b**,**d**). The green border is plant type-like thermo-ASNases associated with functions (**a**,**c**,**d**).

From the viewpoint of a living organism, L-ASNases catalyze a specific biochemical reaction that affects a number of metabolic processes, particularly those necessary for survival of TP/HP microorganisms at high temperatures (Figure 3).

L-ASNases catalyze the conversion of L-asparagine (L-Asn) to L-aspartic acid (or aspartate (Asp)) and ammonia. Asp acts as a critical metabolic hub to interconnect with diverse metabolic pathways (Figure 3a). According to recent data, Asp provides positive effects on mitigating abiotic stresses [60].

First of all, L-ASNases play a vital role in the biosynthesis of aspartate-derived amino acids, namely lysine, threonine, methionine and isoleucine, since aspartic acid is the precursor of these amino acids (Figure 3a) [61,62,63].

According to recent data, one of these amino acid—lysine—is involved in adaptation and tolerance to environmental stresses in various organisms [64,65]. Lysine is a charged amino acid; thus, its accumulation may contribute to the prevention of the protein denaturation caused by high-temperature stress. Due to the NH_2_ groups in the molecule, lysine functions as an ion-coating on the surface of membrane components and proteins to prevent denaturation. Furthermore, lysine is a kosmotropic compound due to the ammonium group in the side chain. The kosmotropic property of lysine may contribute to the protection of cells from the disordering of cellular macromolecules caused by high temperature, similar to the protective effect of compatible solutes against chaotropic stress [64]. As shown in a recent study, the increased level of lysine conferred high-temperature stress tolerance to *E. coli* cells [64].

Lei et al. have shown that Asp is able to enhance heat stress tolerance in plants by up-regulation of a total of nine amino acids—lysine, threonine, glutamate, asparagine, arginine, leucine, valine, glycine, and tryptophan [60].

Beyond its role as an amino acid in proteins, a precursor of amino acids and regulator of amino acid biosynthesis, aspartate is required for conversion of IMP to AMP in de novo purine synthesis and provides the carbon backbone for de novo pyrimidine synthesis (Figure 3a) [66]. The increased content of uracil, UMP, guanosine, and thymine by Asp under heat stress was reported for plants [60]. The authors conclude that Asp may contribute to the maintenance of RNA and DNA synthesis, supporting growth and defense against heat stress [60].

Thus, aspartate deficiency will impair protein, purine nucleotide, and pyrimidine nucleotide synthesis, resulting in decreased cell proliferation. Indeed, Sullivan et al. revealed that exogenous aspartate addition is sufficient to restore proliferation of cells that otherwise stop proliferating or die when activity of electron transport chain is impaired [66].

When excess amounts beyond the normal protein synthesis requirements are available, L-asparagine is hydrolyzed and utilized as a source of carbon and nitrogen [67]. L-ASNases of different types participate in nitrogen and carbon metabolism [35,39]. While expression of bacterial type II L-ASNases involved in carbon metabolism is tightly regulated by different factors [39], bacterial type I L-ASNases, engaged in nitrogen metabolism, appear to be expressed constitutively [30,39]. Asparagine is an important nitrogen storage and transport molecule, due to its relatively high nitrogen-to-carbon ratio (2:4, compared with 2:5 for glutamine, 1:5 for glutamic acid and 1:4 for aspartic acid, for example) and its relative chemical inertia. L-ASNases, participating in recycling the organically bound nitrogen through the ammonification process, play a notable role in nitrogen biogeochemical cycling [68].

In addition to asparaginase activity, some TP/HP L-ASNases have at least residual glutaminolytic activity [6]. Glutamate is known as an important component of several pathways, which links amino acid and respiration metabolism together (Figure 3b) [60]. Glutamate serves as a precursor of proline and arginine [60,69,70]. Arginine, in turn, is the biological precursor of nitric oxide (NO) and polyamines, which significantly contribute to stress tolerance [60,71,72,73]. TP/HP microorganisms require polyamines for growth at high temperatures [74]. Disruption of the genes involved in polyamine synthesis in the hyperthermophile *T. kodakarensis* resulted in severe growth defects at 85 °C, and even more so at 93 °C, which could be slightly reversed at 85 °C but not at 93 °C, by supplying exogenous polyamine spermidine [74].

*T. thermophiles* is also known to produce a variety of polyamines; the most common ones, spermidine and spermine, are synthesised using a distinct pathway from arginine via aminopropyl agmatine [1,71]. Polyamines of *T. thermophilus* were found to be necessary for the maintenance of the ribosome, tRNA^His^, and tRNA^Tyr^ structural integrity during growth at high temperatures [75].

In vitro biochemical studies indicate that polyamines induce structural changes to DNA that are proposed to facilitate growth at extreme temperatures [74,76].

In addition to glutaminase activity, Asp itself can increase accumulation of glutamate and arginine under heat stress. Asp-mediated activation of glutamate metabolism and the TCA enhances heat tolerance [60].

Thus, the direct metabolites of the biochemical reactions catalyzed by L-ASNases—Asp and Glu—are important nodes in the metabolic network of TP/HP microorganisms (Figure 3a,b).

Plant-type L-ASNases identified for TP/HP microorganisms [43,49] are known to have dual L-asparaginase and isoaspartyl aminopeptidase activity [35]. Isoaspartyl aminopeptidase activity protects plants from the spontaneous accumulation of highly toxic β-asparagine dipeptides (isoAsp) [35]. The role of plant-type L-ASNases in mesophilic microorganisms that also possess other types of the enzyme is not clear. Obviously, that is not the case with thermophiles and hyperthermophiles. The rate of isoAsp residue formation increases 910-fold at 90 °C compared with 23 °C [77]. Accumulation of proteins with altered aspartyl- and asparaginyl- residues is believed to be detrimental to cell survival at elevated temperatures [78]; hence, modified or damaged protein (peptide) should be rapidly digested to prevent cellular damage and to provide a potential nutrient source to support cell survival in harsh environments.

According to recent experimental data, heat shock-induced protein aggregates retarded growth of thermophilic bacteria, but expression of β-aspartyl peptidase (BAP) alleviated the growth defect by degrading damaged proteins [78]. BAP can directly hydrolyze isopeptide bonds, resulting in the release of Asp, and this might be an efficient repair mechanism for handing β-aspartyl-containing peptides [78].

Protein homeostasis, a balanced state between folded proteins and protein aggregates, is critical for cellular metabolism and physiology. In extremophiles thriving under harsh environments, in which proteins are vulnerable to protein inactivation and aggregation, cellular protein repair systems play a pivotal role in protein quality control to support cellular integrity and survival [78]. Spontaneous isopeptide bond formation, in particular, isoAsp residues, is accelerated at elevated temperatures [77,79], thus TP/HP L-ASNases with isoaspartyl aminopeptidase activity (plant-type L-ASNases) seems to act as a part of cellular protein repair system important for survival under extremely high temperatures (Figure 3c).

On the other hand, these enzymes may utilize β-aspartyl peptides as a source of amino acids to provide protein biosynthesis under stress conditions, for example, under nutrient depletion.

Another aspect of L-ASNase activity is releasing ammonium ions. Ammonia represents a direct metabolite of the biochemical reaction induced by the enzyme, and exerts profound effects on the electrochemical gradient, membrane potential, and the intracellular pH, thereby affecting the synthesis of ATP and activity of membrane transporters (Figure 3d) [80,81,82]. Singh et al. were the first who reported the influence of L-ASNase on intracellular pH-regulation and H^+^-gradient through ammonia releasing [81].

Ammonia (NH^4+^), the product of the enzymatic hydrolysis of Asn/Gln, could be involved in counteracting the H^+^-influx (acid stress) [81]. Indeed, it is known that the proton and sodium permeabilities of all biological membranes increase with the temperature [83]. The upper temperature border of life depends not only on the stability of the biomolecules, but, in addition, the proton permeability of membranes, which at higher temperatures may become too high to maintain electrochemical proton gradients in order to gain energy [84]. The increased motion of the lipid molecules in the membranes at high temperature causes an increased proton permeability, and in turn, the high proton leakage makes it impossible to control intracellular pH. Among TP/HP microorganisms, TP bacteria have more difficulties to restrict the proton permeation [83].

Thermo-ASNases by releasing ammonium ions can neutralize the excess proton influx, thus maintaining the much required intracellular environment (Figure 3d). The enzyme may be crucial in providing an immediate protective response in maintaining pH and aiding in survival at stress conditions [81]. It is interesting that *E. coli* exploits a similar survival mechanism to maintain an intracellular pH under environmental acidic stress: glutaminase converts Gln to glutamate (Glu) with concomitant release of ammonia, which neutralizes protons, resulting in elevated intracellular pH under acid stress [85].

Overall, these data confirm that thermo-ASNases are key metabolic enzymes, being a part of the complicated heat-tolerance cellular system in TP/HP microorganisms. All products of the enzymatic reaction are associated with multiple metabolic pathways. Playing crucial roles in the integration of cell metabolic systems, they contribute to survival under high temperature stressful conditions.

## 4. Toward a Molecular Understanding of Thermo-ASNases Thermostability

Thermo-ASNases are optimally active at temperatures close to, slightly below, or even above the optimal growth temperature of the host organisms (Table 1). An analysis of the relationships between growth temperatures and optimal catalytic temperatures of enzymes revealed the influence of speciation rather than a common feature. Thus, archaeal thermo-ASNases from *Pyrococcus* sp. have optimum below the growth temperature. Another example is the thermo-ASNase from *A. fulgidus*, an enzyme that has an optimum of activity at 70 °C and the host strain has an optimum of growth at 83 °C.

According to previous data, many individual enzymes in TP and HP have catalytic optima much lower than the growth temperatures [1]. In addition, even the average activity of enzymes isolated from these organisms is 10–20 °C lower than the growth temperature. Extrinsic factors are proposed to provide enzyme adaptation with an optimum below the growth temperature in addition to adaptations in protein sequence and fold [1]. These factors may include compatible solutes such as diglycerol phosphate, found as a mechanism of protein thermostabilization in the hyperthermophiles, in particular, in archaea *A. fulgidus* [86].

Another character of the relationship between growth temperatures and enzyme optima can be observed for *Thermococcus* sp. These archaeal thermo-ASNases display an optimum higher than the growth temperature. It is likely that these enzymes themselves can serve as part of the first-line defense system under stress conditions, including heat stress. More active under elevated temperatures, they can quickly stabilize cellular systems providing an additional margin of safety for host cells.

Thermostability and optimal activity at high temperatures are inherent properties of thermo-ASNases. The differences in thermostability between TP/HP and mesophilic L-ASNases are striking. Previous studies reported that *T. zilligii* L-ASNase showed a slight decrease in activity after incubation for 2 h at 70–85 °C (Table 1) [17]. The half-life of *T. kodakaraensis* L-ASNase and *T. gammatolerans* L-ASNase at 85 °C was more than 120 min [15,87]. Storage of *P. yayanosii* L-ASNase at 37 °C for 1 month showed that 90% of the enzyme activity was retained [20]. The thermostability of non-thermophilic bacterial L-ASNases II is poor compared to thermo-ASNases. When incubated at 70 °C for 30 min, 80% of EcAII activity was lost. The half-life of EcAII at 50 °C (T_(1/2, 50°C)_) was estimated to be 60 min [59]. Mesophilic L-ASNase II from *Bacillus subtilis* retained approximately 14.7% of its activity after 2 h incubation at 50 °C and showed 9.0% residual activity after 2 h incubation at 60 °C [88].

Current studies agree that there is no unique feature or single mechanism responsible for the high functional activity at elevated temperatures and remarkable heat stability of TP/HP proteins [89,90,91]. A concerted action of structural, dynamic and other physicochemical attributes is utilized to ensure the delicate balance between stability and functionality of proteins at such harsh conditions.

### 4.1. Primary Structure Analysis

All proteins, independent of their origin, consist of the 20 canonical amino acids; thus, amino acid composition and the interaction between them in the native TP/HP enzyme structures contribute in their unique characteristics. Correlations between protein thermostability and amino acid composition include a decrease in content of chemically labile residues, a decrease in chain flexibility, an increase in average hydrophobicity, and an increase in frequency of aromatic residues [89,90,91].

**Figure 4:**
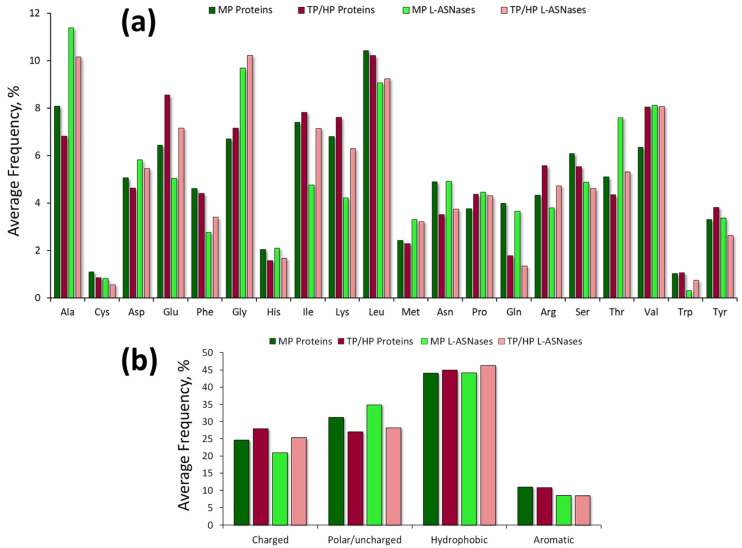
Relative amino acid composition of mesophilic (MP) and thermo-ASNases (thermophilic (TP) and hyperthermophilic (HP)). (**a**) Average amino acid frequencies (%) of MP and thermo-ASNases. (**b**) Average frequency (%) of charged (DEKRH), polar/uncharged (GSTNQYC), hydrophobic (LMIVWPAF), and aromatic (FHWY) amino acid residues in the composition of MP and thermo-ASNases.

The comparison of residue contents in HP/TP and mesophilic L-ASNases revealed more charged residues—Glu, Lys—in the sequences of TP/HP origin (Figure 4a,b). Most thermo-ASNases contain fewer uncharged polar residues (Asn, Gln, His, Cys) and increased residue hydrophobicity (Ile, Val). The same preferential usage of amino acids in thermal adaptation was investigated by comparative proteome analysis, using genomes from mesophiles, thermophiles, and hyperthermophiles (Figure 4) [92].

Camillau and Claverie have reported that thermophilic proteins have less Gln, Ala, and His on their surfaces than mesophilic proteins do and more charged residues on their surfaces, particularly Lys and Glu [93].

With some variation in the content of individual amino acids, the total abundance of charged residues, accompanied by a decrease in the content of polar/uncharged amino acids, is the major feature of thermo-ASNases (Figure 4b).

In general, comparative analysis of the amino acid composition of TP/HP and mesophilic L-ASNases shows trends with variations between and inside these groups (Figure 4, Supplementary Materials, Table S1). For example, cysteine’s high sensitivity to oxidation at high temperature suggests that TP/HP enzymes contain fewer cysteines than their mesophilic counterparts [91]. Thermo-ASNases of hyperthermophilic origin on average contain lower number of cysteine residues (Figure 4a). However, the percentage of Cys residues is higher in the enzymes from thermophilic bacteria (*S. thermoluteus* and *M. roseus*) (Supplementary Materials, Table S1).

Initially, it was believed that susceptibility of cysteines and disulfide bridges to destruction at high temperatures determined the upper limit for the stability of proteins containing disulfide bridges [91]. Nevertheless, recent observations indicate, cysteines that are present in proteins from hyperthermophiles are often involved in specific stabilizing interactions (e.g., disulfide bridges and metal liganding) and/or are inaccessible to the solvent [91]. Disulfide bridges can serve as a stabilization strategy, and conformational environment and solvent accessibility are determining factors in the protection of disulfide bridges against destruction. Thermo-ASNase from hyperthermophilic archaea *P. furiosus* PfA that grows at optimum temperature of 100 °C confirms the fact that not all disulfide bridges have equal susceptibility to destruction. PfA contains two cysteines (Supplementary Materials, Table S1) and displays stability in harsh conditions, including denaturation at 8 M concentration of urea [24].

It is worth mentioning that elevated temperatures trigger not only cysteine oxidation, but also such spontaneous chemical modifications as deamidation. Two deamidation mechanisms are known for Asn and Gln residues [91]. Thus, as part of the adaptation mechanism against deamidation at high temperatures, some TP/HP enzymes contain less Asn and Gln [91,94]. These amino acids are also less common in thermo-ASNases (Figure 4a).

In summary, the results of the primary structure analysis of thermo-ASNases support the conclusion that thermophilic adaptation cannot be defined only in terms of significant differences in the amino acid composition [91]. Probably, the distribution of the residues, their positions and interactions can affect the thermoactivity and thermostability of TP/HP proteins to a greater extent.

### 4.2. Structural Features and Key Residues Essential for Thermoactivity and Thermostability of Thermo-ASNases

Elucidation of the available L-ASNase crystal structures from hyperthermophilic archaea *P. horikoshii*, *P. furiosus*, and *T. kodakaraensis* in comparison with the enzymes from mesophiles revealed increased content of salt bridges in thermo-ASNases—17.5 ÷ 20.0% vs. 9.2 ÷ 13.9% for the non-thermophilic enzymes [14]. While no significant differences were observed between these groups in the number of hydrogen bonds in helix structures t [14].

According to previous studies, most thermophilic proteins tend to have more salt bridges than their mesophilic homologs [95,96,97].

Chan et al. demonstrated that stabilizing salt bridges enhance the thermostability of proteins by reducing the heat capacity change in unfolding ΔCp. In particular, the double-mutant-cycle approach revealed that breaking a salt bridge increased the ΔCp value, and conversely, the formation of each salt-bridge contributed to a reduction in ΔCp by 0.8–1.0 kJ mol^−1^ K^−1^. A decrease in ΔCp due to the extra salt bridges in thermophilic proteins, in turn, enhances protein thermostability by up-shifting and broadening the protein stability curve so that the protein remains stable at a wider range of temperatures [97].

An in-depth evaluation of the role of distinct inter-residue interactions and individual amino acid contributions for maintaining thermoactivity and thermostability of thermo-ASNases was carried out by Li et al. [59]. The rationale for the difference in thermostability of the thermophilic and non-thermophilic L-ASNases was based on the comparative analysis of the representative enzymes from *P. yayanosii* CH1 (PyA), *T. gammatolerans* (TgA), *B. subtilis* (BsAII), and *E. coli* (EcAII).

#### 4.2.1. Identification of Amino Acid Residues Essential for Thermoactivity and Thermostability of Thermo-ASNases

Despite significant divergence in amino acid sequences of L-ASNases, the architecture and composition of their active-site pockets are highly conserved [38,59,98]. So-called “special residues” are located near to the highly conserved regions in thermo-ASNases—highly conserved amino acid residues different from that of non-thermophilic L-ASNases.

**Figure 5:**
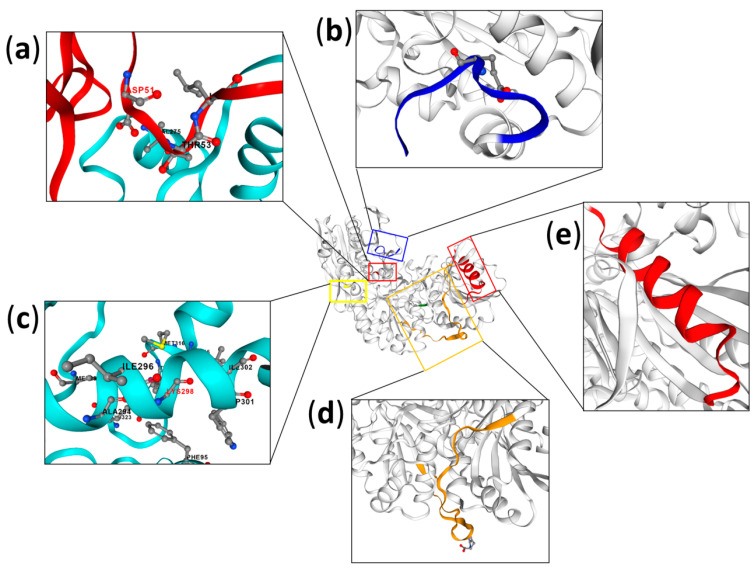
Key residues and structural features essential for thermoactivity and thermostability of thermo-ASNases. Structures are built in SWISS-MODEL (http://swissmodel.expasy.org/ accessed on 13 January 2023). (**a**,**c**) “Special residues” of thermo-ASNase *P. yayanosii* (PyA)—D51 and K298, respectively. (**b**) C-terminal residues of PyA. (**d**) Linker and β-strand residues L179-V-V-N182 of the N-terminal domain of thermo-ASNase from *P. furiosus*. (**e**) α3 secondary structure of PyA thermo-ASNase.

Analysis of PyA “special residue” mutants with respect to their shift in optimum temperature, activity, and the values of half-life revealed two of the nine substitutions analyzed that affect the thermostability and optimum temperature of the enzyme—D51G and K298L (Figure 5a,c). The half-lives at 85 °C (T_(1/2, 85 °C)_) of mutants D51G and K298L were 70 min and 55 min lower than those of the wild type, respectively. D51G and K298L had 30 °C and 10 °C reduction in optimum temperature compared to PyA, respectively (Table 2) [59].

Residue D51 is located on a loop around the active site. The mutation D51G reduced thermostability of the enzyme due to the deficiency of two polar contacts with the surrounding residues, which led to an increase in the surface charge near the active site [59].

Unlike residue D51, K298 was located at the core of the α-helix (residues 291–305) far away from the active site [59]. Compared with mutated residue K298L, residue K298 not only formed polar contacts with the residues (A294, T297, I302) in the α-helix (residues 291–305) but also formed contacts with C terminal residues (L315, M316, T318, and E233). The mutated residue K298L led to a decrease in C terminal connectivity and resulted in decreased thermostability. Thus, K298 represents one of key residues responsible for the thermostability of thermo-ASNase. A tight junction at the C terminal is essential for maintaining high thermal stability of the enzyme [59].

Analysis of conformational dynamics revealed that both mutations reduced overall tightness of the protein structure that resulted in decreased thermostability of the enzyme.

The obtained results were confirmed using thermo-ASNase from archaea *T. gammatolerans*. Mutation of the corresponding residues D52G and K298L showed decreased T_(1/2, 85 °C)_ (Table 2) [59]. In addition, the mutated enzymes D52G and K298L displayed a lower optimum temperature, though to a different extent (Table 2).

Since the “special” residues D51 and K298 of PyA (corresponding to residues D52 and K298 of TgA, respectively) are highly conserved in 1000 homologous enzymes, they can be considered essential to maintaining thermoactivity and thermostability of a large number of thermo-ASNases.

These findings were confirmed in the reverse experiments using EcAII and BsAII. Corresponding to the thermo-ASNase mutations D−G and K−L, the mutated forms EcA II G57D and L305K showed a higher thermostability, with the half-life at 50 °C (T_(1/2, 50 °C)_) 40 min and 30 min longer than that of EcAII, respectively (Table 2). Improved thermostability was also revealed after substitutions at the corresponding residues G107D and L354K of BsAII [59].

The results suggest that “special” residues D51 and K298 of PyA (corresponding to D52 and K298 of TgA, G57 and L305 of EcAII, G107 and L354 of BsAII) make L-ASNases adapted to different working temperatures.

#### 4.2.2. Key Secondary Structures Associated with the Thermal Adaptation of Thermo-ASNases

Not only amino acid residues affect the enzyme characteristics, but also the secondary structures. In attempts to identify the key secondary structures responsible for thermostability of the thermo-ASNase PyA, the secondary structures near the highly conserved and “special” residues—PyA-β, PyA-loop, PyA-α1, PyA-α2, PyA-α3, and PyA-α4—were replaced with the corresponding structure of the mesophilic EcAII [59].

While PyA-loop, PyA-α1, PyA-α2, and PyA-α4 were lethal and PyA-β showed no significant influence on activity and stability under high temperatures, the mutation PYA-α3 (Figure 5e) showed a lower optimum temperature and shorter T_(1/2, 85 °C)_ compared to the wild-type enzyme (Table 3). Based on the protein structure model, replacing of α3 resulted in the cleavage of the helical structure and a decrease in three polar contacts with the surrounding amino acid residues, resulting in the formation of a more flexible structure. Moreover, the mutant had more positive surface charge and increased the root mean square fluctuation (RMSF) [59]. These results indicated that flexibility of the structure coupled with high positive surface charge decreased thermal stability of PyA thermo-ASNase after structural mutation.

However, two additional polar contacts with the surrounding residues appeared in the mutated structure α3 of TgA [59]. Together with a reduced positive surface charge, these changes resulted in improved thermostability compared to the wild-type enzyme (Table 3).

Thus, it was revealed, that α3 (Figure 5e) exerted a great impact on the thermostability of thermo-ASNases, though the effect can be different depending on the character of interaction with the surrounding amino acid residues.

#### 4.2.3. The Role of C-Terminal Amino Acid Residues in Thermo-ASNase Thermostability

Leng et al. emphasized that thermophilic proteins tend to insert or delete residues in the C-terminal domain [99]. The C-terminal domain has been shown to play a vital role in the stabilization of the structure and is proposed to contribute to the thermostability of thermophilic proteins [99,100].

Thermo-ASNases have extended C-terminal region with additional 8–9 amino acid residues compared to mesophilic L-ASNases II. Considering that the C-terminal residues often display a significant impact on the level of protein thermostability, truncated forms of thermo-ASNases were obtained and analyzed [59]. Li et al. have found that the C-terminal residues affected thermostability of thermo-ASNase from *P. yayanosii* CH1 (PyA), but not from *T. gammatolerans* [59]. PyA-ΔC9 (Figure 5b) displayed a 45 min shorter half-life (T_(1/2, 85°C)_) with minimal change in optimal temperature.

Analysis of the mutants PyA-Δ1–PyA-Δ8 revealed that the 6th C-terminal residue of PyA—E323—affects the thermostability of thermo-ASNase. Truncation of 6 C-terminal residues reduced T_(1/2, 85°C)_ from 105 min for the wild type to 60 min for the mutant [59]. E323 is probably a key residue for the thermal stability of PyA, which is involved in multiple interactions with C-terminal loop and core residues. The loss of connection between the C-terminal and the helix (residues 292–305) after mutation led to protein instability [59].

Thus, the C-terminal residues were identified as a structural determinant of PyA thermo-ASNase thermal stability.

#### 4.2.4. The Role of Amino Acid Residues of the N-Terminal Domain in Thermo-ASNase Thermostability

Searching possibilities to improve characteristics of thermo-ASNase from *P. furiosus* PfA, Sharma et al. have obtained conjoined functional version of cPfA, lacking a 19-residue long linker which connects the two domains, namely the N- and C-halves (N-PfA and C-PfA, respectively) (Figure 5d) [101].

The cPfA displayed improved specific activity and similar thermophilic profile compared to the wild type full-length protein PfA. At 80 °C the specific activity was increased from 338 U/mg for PfAwt to 413 U/mg for cPfA [102]. However, further cutting of the terminal short β-strand in the N-terminal domain revealed that this structure is important in providing stability under high temperature conditions. For the resulting defunct form dcPfA (lacking both linker and LVVN peptide), an increase in temperature triggered the formation of high molecular weight aggregates—assemblies with non-specific oligomerization sites—leading to a decrease in stability of the protein [101]. The most significant decrease in dcPfA activity was observed at temperatures above 50 °C. The loss of activity for dcPfA was 38%, 60%, 80% and 90% compared to cPfA, at 45 °C, 60 °C, 70 °C and 80 °C, respectively.

Thus, short β-strand residues L179-V-V-N182 of the N-terminal domain affect thermostability of PfA Supplementation of the peptide LVVN to the defunct enzyme restored structural framework with mesophile-type functionality.

### 4.3. Contribution of the Oligomeric State to the Thermostability of Thermo-ASNases

Oligomerization is recognized as a mechanism for protein stabilization to confer a thermophilic adaptation [103]. In general, proteins isolated from thermal environments are oligomers that have large hydrophobic cores and increased electrostatic interactions, disulfide bonds, salt bridging, and surface charges [104].

Since L-ASNases are active only in the oligomeric form, oligomerization is a key process in formation of the active site structure of this enzyme and its substrate specificity. Most mesophilic L-ASNases, including EcA, EwA, L-ASNases of *Helicobacter pylori*, *Pectobacterium carotovorum*, exist as homotetramers with a molecular mass of 140–150 kDa [6]. In contrast to mesophilic counterparts, thermo-ASNases, in particular, known hyperthermophilic L-ASNases, are supposed to exist in dimeric form (at ambient temperatures) [6].

In the crystallographic study of thermo-ASNase from hyperthermophilic archaea *T. kodakaraensis* KOD1—TkA—it was revealed that each subunit of the enzyme consists of an N-terminal and a C-terminal α/β domain connected by a short linker region [14]. The larger N-terminal domain is formed by eight β-sheet folds (β1, β4–8, β11, β12) and four α-helices (α1–4). It has been shown that the β-hairpin composed of strands β2 and β3 is highly flexible. This β-hairpin is involved in substrate binding and catalysis, and adopts “open” or “closed” conformation by TkA subunits [14].

The relatively small C-terminal domain is formed mainly by a three-stranded parallel β-sheet (β13–β15) and five α–helices (α5–α9) [14].

The substrate specificity and activity of L-ASNases are directly related to the oligomerization process. TkA is active only in the dimeric form (in low temperature studies) [14]. Substrate recognition and catalytic activity involve both subunits of the enzyme, particularly, Thr11, Tyr21, Ser54, Thr55, Thr85, Asp86 and Lys156, as well as Tyr233 and Glu275, which are highly conserved for L-ASNases [14].

#### 4.3.1. Folding, Association, and Intersubunit Interactions as Part of Thermo-ASNase Thermostability

Particular interest in the oligomerization of L-ASNases from microorganisms living at elevated temperatures is associated not only with the fact that oligomerization determines the enzyme activity (active sites are located in the contact zone of subunits), but also with the fact that for proteins with high hydrophobicity at elevated temperatures the formation of aggregates during assembly is highly probable; in this context, the oligomerization mechanism can be an evolutionary adaptation, which helps to avoid accumulation of these aggregates [22,23].

Using thermo-ASNase from archaea *P. furiosus* PfA as a model system representing homodimeric (at ambient temperatures) L-ASNase of hyperthermophilic origin, quaternary structure formation has been studied [22,23]. It has been shown that the more stable N-terminal domain plays a decisive role in the assembly of PfA. The process operates sequentially: the part of the polypeptide chain that forms the N-terminal domain folds and forms a stable core, after which the folding of the C-terminal domain occurs. For PfA asparaginase, folding and association of domains are coupled, which leads to high kinetic stability and prevents aggregation [22,23].

The N-domain of PfA acts like a folding scaffold and assists the folding of remaining polypeptide [23]. The coupled process of folding and oligomerization apparently represents the evolutionary adaptation that avoids accumulation of partially folded monomeric intermediates with a high probability of aggregation at elevated temperatures.

Thermal stability and high activity at elevated temperatures are explained by a strong intersubunit interaction. The structural analysis of the PfA enzyme showed that the monomers are assembled in a head-to-tail fashion, where the N-terminal domain of the monomer (NPfAI) is linked to the C-terminal domain (CPfAII) [23]. The two C-domains, CPfAI and CPfAII, were found to align centrally. Analysis revealed that for individual domains the intra-monomeric H-bonding and hydrophobic interactions are more as compared to intermonomeric domain-wise interactions. When all the inter-monomeric interactions are combined (NPfAI-CPfAII, CPfAI-CPFAII and NPfAII-CPfAI), they far exceeded the intra-monomeric interactions. The numbers of salt bridges in the contact zone between the monomers were also found to exceed their number inside the NPfA-CPfA monomer [23]. Overall data suggest that the extreme thermal stability of PfA is due to the presence of high intersubunit associative forces supported by extensive H-bonding and ionic interactions network.

Analyzing thermostable proteins, Reed et al. highlighted tighter packing of the hydrophobic core of TP proteins due to favorable hydrophobic interactions and increased number of ionic interactions as one of the most prominent factors of thermal adaptation to retain structure and function under extreme temperatures [104].

#### 4.3.2. Heat-Induced Thermo-ASNase Super-Stable Assembly

The reduction in the oligomeric state can abolish the high thermostability of enzymes [105]. As rule, a higher oligomeric state is favorable for TP proteins [104]. In this view, it is interesting that the biological assembly of both type I and II L-ASNases of mesophilic origin is a homotetramer, whereas most of the known hyperthermophilic L-ASNases are supposed to exist in dimeric form [6]. The results of Sharma et al. shed some light on the situation: most of the structural information on thermo-ASNase was obtained from low temperature studies; in fact, at elevated temperatures, the formation of higher order oligomeric states of thermo-ASNase occurs [101].

As relevant shape–function information cannot be extrapolated from partial low temperature data, Sharma et al. have performed the high temperature small-angle X-ray scattering (SAXS) experiments along with enzyme assay to obtain new information on the structural organization of thermo-ASNase from *P. furiosus* PfA at higher temperatures [101]. Analysis revealed that PfA remains predominantly dimer (about 70 kDa) from 25 to 50 °C. Upon further increase in temperature, the molecular masses of particles in solution were clearly bigger than dimer, and at 80 °C the estimated mass was closer to that of tetramer of PfA. The experiments indicated that though the dimer existed at ambient temperature, heating induced higher order association.

Based on the available crystal structures and interaction analysis, 4 hydrogen bonds, 2 salt bridges and 44 hydrophobic interactions between domains were revealed for PfA tetramer [101]. Most of the residues involved in interactions belong to the terminal region of the N-terminal domain depicting that this region might be involved in molecular associations.

### 4.4. The Possible Role of Substrate Binding on the Oligomerization Properties of Thermo-ASNases

Substrate binding can also play a role in oligomerization and stabilization of thermo-ASNases at elevated temperatures.

The hypothesis of substrate-induced oligomerization switch was first proposed for thermo-ASNases by Sharma et al. [101]. Analysis of the crystal structures of the enzyme from *P. furiosus* PfA and its form cPfA, lacking a 19-residue long linker which connects N-, C-domains (Figure 5d), revealed, that the unstructured active-site loop in ligand-free enzyme became structured once the active site was occupied by the ligand [102]. The structured active sites of adjoining protein molecules established contact through H-bonds and hydrophobic interactions [101]. The number of interactions between protein molecules was approximately double, indicating that they were capable of forming more stable assemblies in the presence of substrate (Table 4) [101]. Thus, the authors concluded that although thermo-ASNase PfA existed in an oligomeric state under high temperature conditions, it is more likely that conformational changes in the presence of substrate significantly enhanced its tendency to form stronger interactions and higher order associations [101].

The influence of substrate binding on the oligomerization properties of different proteins was reported previously [106,107,108]. As an example, it was revealed that substrate stabilizes dihydrodipicolinate synthase by locking the conformation of the tight dimer unit, thus promoting docking of the tight dimers together to yield the more active tetrameric form [106]. According to the crystal structure analysis, substrate binding promoted greater contacts at the dimerization interface, which essentially reduced the conformational flexibility of the dimer; that, in turn, promoted tetramerization [106]. Moreover, it was associated with an increase in the stability of the enzyme. In the thermal denaturation experiments the apparent melting temperatures increased from 63 °C in substrate-free form to 74 °C in the presence of the substrate. An increased thermal stability was confirmed to be specific to the substrate, but no effect was observed in the presence of substrate analogues [106].

Lactate dehydrogenase was also reported to undergo an obvious conformational change upon substrate binding and stabilized against thermal denaturation by its substrates, NADH and lactate [109].

In summary, until recently nothing was known about the ability of substrate binding to stabilize thermo-ASNases at elevated temperatures. A novel mechanism for ligand-induced stabilization revealed for the enzyme from hyperthermophilic archaea *P. furiosus* offers insight into the synergy between quaternary structure, protein dynamics, and catalytic function for adaptation to hot environments.

## 5. Conclusions

The research interest in L-ASNases is primarily due to their wide application in biotechnology. In the search for forms with improved efficacy—enhanced activity and stability—many microbial species, from archaeal, bacterial and eukaryotic sources, have been screened for L-ASNase production. Among them, thermophiles and hyperthermophiles produce L-ASNases with extraordinary properties—stable and heat resistant thermo-ASNases with superior performances for biotechnology.

In writing this review we have attempted to present a broader view of the thermo-ASNases: from the types currently identified, to highlighting striking differences from mesophilic counterparts, proposal for their classification, summary of their physiological role as a part of the complicated heat-tolerance cellular system in TP/HP microorganisms, and interpretation their thermostability and optimal activity at high temperatures at the molecular scale.

Nature created ingenious molecular mechanisms for maintaining thermoactivity and thermostability of thermo-ASNases, so that they protect their host from extreme heat.

Obviously, the fact that TP/HP usually possess a set of L-asparaginases in the genomes, and these enzymes are expressed at a high level, is not surprising in view of the predicted role of thermo-ASNases in the integration of cell metabolic systems for survival under high temperatures.

Getting insight into the mechanisms used by thermo-ASNases to modulate their thermoresistance is a way to better understand how TP/HP adapt to these extreme environments. The results obtained so far confirm that no single mechanism is responsible for the remarkable stability of thermo-ASNases. All levels of adaptation—from amino acid composition to structural features, protein dynamics, and catalytic function—provide synergy. The findings summarized in the review may pave the way to rationally engineer both thermo-ASNases suitable for special application at moderate temperatures and their mesophilic counterparts with enhanced stability. This can revolutionize their future-oriented applications.

## Figures and Tables

**Table 1:** Summary of thermo-ASNases: producing microorganisms, types, main characteristics.

Microorganism—Source of Thermo-ASNase	Growth Temperature Range/ Optimum Growth, °C	L-Asparaginase Abbreviation	Type	Oligomeric State	Optimum t, °C	Specific Activity, U/mg	Kinetic Parameters	Thermostability	Refs.
Hyperthermophilic Archaea									
*Thermococcus kodakaraensis* KOD1	60–100/85	TkAI	Bacterial-type I	Homodimer	90.0	978.7	K_m_ = 2.6 mM V_max_ = 1121 μmol min^−1^ mg^−1^	90 °C: retained 90% of its activity after 32 h	[14,16,42]
		TkAI	Bacterial-type I	Homodimer	85.0	2350	K_m_ = 5.5 mM V_max_ = 3300 μmol min^−1^ mg^−1^	Half-life at 85 °C—130 min; boiling water—14 min	[15]
		TkAIII	Plant-type	Dimer	85.0	767	K_m_ = 3.1 mM V_max_ = 833 μmol min^−1^ mg^−1^	Half-life of the enzyme was nearly 18 h at 85 °C	[43]
*Thermococcus zilligii*	75–80/75	TzAI	Bacterial-type I	Homodimer	90.0	5278 ± 32	K_m_ = 6.08 mM	85 °C: retained 70% of its activity after 2 h	[17]
*Thermococcus gammatolerans*	55–95/88	TgAI	Bacterial-type I	Homodimer	85.0	7622	K_m_ = 10.0 mM	70–95 °C: retained more than 60% of its initial activity after 0.5 h; 95 °C—43% of its activity after 2 h	[18]
*Thermococcus sibiricus*	40–88/78	TsAI	Bacterial-type I	Homodimer	90.0	2164	K_m_ = 2.8 mM V_max_ = 1200 μmol min^−1^	90 °C: retained 86% of its initial activity after 20 min	[19]
*Pyrococcus yayanosii* CH1	80–108/98	PyAI	Bacterial-type I	Homodimer	95.0	1483.81	K_m_ = 6.5 mM V_max_ = 2929 μmol min^−1^	70, 80, 85, 90, 95 °C: retained 57%, 50%, 47%, 40%, and 35% of its activity after 2 h of exposure, respectively. A half-life at 85 °C—105 min.	[20]
*Pyrococcus furiosus*	70–103/100	PfAI	Bacterial-type I	Homodimer	~85.0	550	K_m_ (80 °C) = 12 mM	No data	[44]
*Pyrococcus abyssi*	67–102/96	PaAI	Bacterial-type I	Homodimer	80.0	1175	K_m_ = 2.05 mM	Active in a wide range of temperature levels—40–100 °C. Retained 100% of its activity when incubated at 80 °C for 5 min	[26]
*Pyrococcus horikoshii*	80–102/98	PhAI	Bacterial-type I	Homodimer	No data	No data	No data	No data	[25]
*Archaeoglobus fulgidus*	60–95/83	AfA	No data	Homodimer	70.0		K_m_ (70 °C) = 0.005 mM	Retained 70% of its activity after 5 min incubation at 85 °C, and ~10% of its activity at 95 °C. Retained ~90% of its activity after a four-month storage at room temperature	[27]
*Pyrobaculum calidifontis*	80–98 (able to grow at 75–100 °C)/90–95	PcAIII	Plant-type	Dimer	≥100		K_m_ = 4.5 ± 0.4 mM V_max_ = 355 ± 13 μmol min^−1^ mg^−1^	Exhibited a half-life of 150 min in the boiling water	[45]
Thermophilic Bacteria									
*Streptomyces thermoluteus* subsp. fuscus NBRC 14270	Growth temperature ranges of thermophilic *Streptomyces* sp.: 28–68 °C	StAII	Bacterial-type II	Homodimer	63.6	68.09	K_m_ = 1.83 mM V_max_ = 92.73 μmol min^−1^ mg^−1^	No data	[46]
*Thermus thermophilus*	47–85/65–72	TtAII	Bacterial-type II	Hexamer	≥70	840	K_m_ = 2.8 mM	No data	[47,48]
*Melioribacter roseus*	35–60/52–55	MrAII	Bacterial-type II	No data	70	1530	K_m_ = 1.4 mM V_max_ = 5573 μmol min^−1^	Retained 70% of its initial activity after 60 min incubation at 40 °C	[49]
*Thermus aquaticus*	40–79/70	TaA	No data	Monomer	75.0	585	K_m_ = 8.6 mM	The half-life of the enzyme at 85 °C was 40 min	[50]

**Table 2:** Amino acid residues affected thermoactivity and thermostability of thermo-ASNases [59,98].

Enzyme	Variants	Optimum Temperature, °C	T_(1/2)_ *, min	Specific Activity, U/mg
PyA	Wild type	95	105 ± 5	1483 ± 63
	D51G	65	35 ± 4	341 ± 29
	K298L	85	50 ± 4	1513 ± 61
TgA	Wild type	85	135 ± 5	5381 ± 97
	D52G	60	55 ± 3	962 ± 51
	K298L	80	75 ± 4	5310 ± 93
EcAII	Wild type	37	60 ± 5	235 ± 21
	G57D	50	100 ± 4	643 ± 33
	L305K	45	90 ± 5	243 ± 17
BsAII	Wild type	40	75 ± 5	92 ± 4.1
	G107D	40	**	182.84 ± 7.49
	L354K	45	105 ± 3	101 ± 5.2

* T_(1/2)_ of thermo-ASNases PyA and TgA and their mutations were determined at 85 °C. T_(1/2)_ of EcAII, BsAII and their mutations were determined at 50 °C. **—half-inactivation temperature Tm was determined: BstAII wild type—46.8 °C, G107D—64.3 °C [98].

**Table 3:** α3 affects thermostability of thermo-ASNases [59].

Enzyme	Variants	Optimum Temperature, °C	T_(1/2, 85°C)_, min	Specific Activity, U/mg
PyA	Wild type	95	105 ± 5	1483 ± 63
	α3	80	50 ± 3	1360 ± 59
TgA	Wild type	85	135 ± 5	5381 ± 97
	α3	90	155 ± 7	5113 ± 84

**Table 4:** The number of interactions in different crystal structures of *P. furiosus* thermo-ASNase [101].

Enzyme	Conditions	No. of Salt Bridges	No. of H-Bonds	No. of Hydrophobic Interactions
PfA	With ligand	2	4	44
cPfA	With ligand, 18 °C	0	10	83
	With ligand, 37 °C	2	8	86
	Ligand-free	1	3	48

## Data Availability

Not applicable.

## Supplementary Materials

The supporting information can be downloaded at: https://www.mdpi.com/article/10.3390/ijms24032674/s1.

## References

[1] Engqvist M.K.M. (2018). Correlating enzyme annotations with a large set of microbial growth temperatures reveals metabolic adaptations to growth at diverse temperatures. BMC Microbiol..

[2] Stetter K.O. (1996). Hyperthermophilic Procaryotes. FEMS Microbiol. Rev..

[3] Imanaka T. (2011). Molecular bases of thermophily in hyperthermophiles. Proc. Jpn. Acad. Ser. B.

[4] Lopes A.M., de Oliveira-Nascimento L., Ribeiro A., Tairum C.A., Breyer C.A., de Oliveira M.A., Monteiro G., de Souza-Motta C.M., de Magalhães P.O., Avendaño J.G.F. (2017). Therapeutic L-Asparaginase: Upstream, Downstream and Beyond. Crit. Rev. Biotechnol..

[5] Nunes J.C.F., Cristóvão R.O., Freire M.G., Santos-Ebinuma V.C., Faria J.L., Silva C.G., Tavares A.P.M. (2020). Recent Strategies and Applications for L-Asparaginase Confinement. Molecules.

[6] Dumina M.V., Eldarov M.A., Zdanov D.D., Sokolov N.N. (2020). L-Asparaginases of Extremophilic Microorganisms in Biomedicine. Biochem. (Moscow) Suppl. Ser. B Biomed. Chem..

[7] de Oliveira Lima I.G., Bispo J.R.S., da Silva M.B., de Oliveira Feitosa A., dos Santos A.C.M., Moreira M.S.A., Passarini M.R.Z., Câmara P.E.A.S., Rosa L.H., Oliveira V.M. (2021). Technological Prospecting: Mapping Patents on L-asparaginases from Extremophilic Microorganisms. Recent Patents Biotechnol..

[8] Mahajan R.V., Kumar V., Rajendran V., Saran S., Ghosh P.C., Saxena R.K. (2014). Purification and Characterization of a Novel and Robust L-Asparaginase Having Low-Glutaminase Activity from Bacillus licheniformis: In Vitro Evaluation of Anti-Cancerous Properties. PLoS ONE.

[9] Ali U., Naveed M., Ullah A., Ali K., Shah S.A., Fahad S., Mumtaz A.S. (2016). L-asparaginase as a critical component to combat Acute Lymphoblastic Leukaemia (ALL): A novel approach to target ALL. Eur. J. Pharmacol..

[10] Muso-Cachumba J.J., Antunes F.A.F., Peres G.F.D., Brumano L., Santos J., Da Silva S.S. (2016). Current applications and different approaches for microbial l-asparaginase production. Braz. J. Microbiol..

[11] National Toxicology Program (2019). Report on Carcinogens.

[12] Verma N., Kumar K., Kaur G., Anand S.E. (2007). *coli* K-12 Asparaginase-Based Asparagine Biosensor for Leukemia. Artif. Cells Blood Substit. Biotechnol..

[13] Kumar K., Kataria M., Verma N. (2012). Plant asparaginase-based asparagine biosensor for leukemia. Artif. Cells Nanomed. Biotechnol..

[14] Guo J., Coker A.R., Wood S.P., Cooper J.B., Chohan S.M., Rashid N., Akhtar M. (2017). Structure and Function of the Thermostable L-Asparaginase from Thermococcus Kodakarensis. Acta Cryst..

[15] Chohan S.M., Rashid N. (2013). TK1656, a thermostable l-asparaginase from Thermococcus kodakaraensis, exhibiting highest ever reported enzyme activity. J. Biosci. Bioeng..

[16] Hong S.-J., Lee Y.-H., Khan A.R., Ullah I., Lee C., Park C.K., Shin J.-H. (2014). Cloning, expression, and characterization of thermophilicL-asparaginase from *Thermococcus kodakarensis* KOD1. J. Basic Microbiol..

[17] Zuo S., Zhang T., Jiang B., Mu W. (2015). Reduction of acrylamide level through blanching with treatment by an extremely thermostable l-asparaginase during French fries processing. Extremophiles.

[18] Zuo S., Xue D., Zhang T., Jiang B., Mu W. (2014). Biochemical characterization of an extremely thermostable l-asparaginase from Thermococcus gammatolerans EJ3. J. Mol. Catal. B Enzym..

[19] Dumina M., Zhgun A., Pokrovskaya M., Aleksandrova S., Zhdanov D., Sokolov N., El’Darov M. (2021). A Novel L-Asparaginase from Hyperthermophilic Archaeon *Thermococcus sibiricus*: Heterologous Expression and Characterization for Biotechnology Application. Int. J. Mol. Sci..

[20] Li X., Zhang X., Xu S., Zhang H., Xu M., Yang T., Wang L., Qian H., Zhang H., Fang H. (2018). Simultaneous cell disruption and semi-quantitative activity assays for high-throughput screening of thermostable L-asparaginases. Sci. Rep..

[21] Bansal S., Srivastava A., Mukherjee G., Pandey R., Verma A.K., Mishra P., Kundu B. (2012). Hyperthermophilic asparaginase mutants with enhanced substrate affinity and antineoplastic activity: Structural insights on their mechanism of action. FASEB J..

[22] Garg D.K., Kundu B. (2017). Hyperthermophilic l -asparaginase bypasses monomeric intermediates during folding to retain cooperativity and avoid amyloid assembly. Arch. Biochem. Biophys..

[23] Garg D.K., Tomar R., Dhoke R.R., Srivastava A., Kundu B. (2015). Domains of Pyrococcus furiosus l-asparaginase fold sequentially and assemble through strong intersubunit associative forces. Extremophiles.

[24] Bansal S., Gnaneswari D., Mishra P., Kundu B. (2010). Structural stability and functional analysis of L-asparaginase from Pyrococcus furiosus. Biochemistry.

[25] Yao M., Yasutake Y., Morita H., Tanaka I. (2005). Structure of the type IL-asparaginase from the hyperthermophilic archaeon *Pyrococcus horikoshii* at 2.16 Å resolution. Acta Crystallogr. Sect. D Biol. Crystallogr..

[26] Nadeem M.S., Khan J.A., Al-Ghamdi M.A., Khan M.I., Zeyadi M.A. (2022). Studies on the recombinant production and anticancer activity of thermostable L- asparaginase I from Pyrococcus abyssi. Braz. J. Biol..

[27] Li J., Wang J., Bachas L.G. (2002). Biosensor for Asparagine Using a Thermostable Recombinant Asparaginase from *Archaeoglobus fulgidus*. Anal. Chem..

[28] Chohan S.M., Rashid N., Sajed M., Imanaka T. (2018). Pcal_0970: An extremely thermostable l-asparaginase from Pyrobaculum calidifontis with no detectable glutaminase activity. Folia Microbiol..

[29] Sajed M., Naeem S.U., Rashid N. (2021). l-Asparaginases from hyperthermophilic archaea and their applications. Microbial Extremozymes.

[30] Loch J.I., Jaskolski M. (2021). Structural and biophysical aspects of L-asparaginases: A growing family with amazing diversity. Iucrj.

[31] Jia R., Wan X., Geng X., Xue D., Xie Z., Chen C. (2021). Microbial L-asparaginase for Application in Acrylamide Mitigation from Food: Current Research Status and Future Perspectives. Microorganisms.

[32] Bath De Morais S., de Souza T.D.A.C.B. (2021). Human L-asparaginase: Acquiring knowledge of its activation (Review). Int. J. Oncol..

[33] Dumina M., Eldarov M., Zdanov D., Sokolov N. (2020). L-asparaginases of extremophilic microorganisms in biomedicine. Biomeditsinskaya Khimiya.

[34] Prahl A., Pazgier M., Hejazi M., Lockau W., Lubkowski J. (2004). Structure of the isoaspartyl peptidase withL-asparaginase activity from *Escherichia coli*. Acta Crystallogr. Sect. D Biol. Crystallogr..

[35] Michalska K., Jaskolski M. (2006). Structural aspects of L-asparaginases, their friends and relations. Acta Biochim. Pol..

[36] Nomme J., Su Y., Konrad M., Lavie A. (2012). Structures of Apo and Product-Bound Human l-Asparaginase: Insights into the Mechanism of Autoproteolysis and Substrate Hydrolysis. Biochemistry.

[37] Su Y., Karamitros C.S., Nomme J., McSorley T., Konrad M., Lavie A. (2013). Free Glycine Accelerates the Autoproteolytic Activation of Human Asparaginase. Chem. Biol..

[38] Lubkowski J., Wlodawer A. (2021). Structural and biochemical properties of L-asparaginase. FEBS J..

[39] Sharafi Z., Barati M., Khoshayand M., Adrangi S. (2017). Screening for Type II L-Asparaginases: Lessons from the Genus Halomonas. Iran. J. Pharm. Res..

[40] Izadpanah Qeshmi F., Homaei A., Fernandes P., Javadpour S. (2018). Marine microbial L-asparaginase: Biochemistry, molecular approaches and applications in tumor therapy and in food industry. Microbiol. Res..

[41] Bejger M., Imiolczyk B., Clavel D., Gilski M., Pajak A., Marsolais F., Jaskolski M. (2014). Na^+^/K^+^ exchange switches the catalytic apparatus of potassium-dependent plantL-asparaginase. Acta Crystallogr. Sect. D Biol. Crystallogr..

[42] Uehara R., Takano K., Kanaya S., Koga Y. (2016). Hyperthermophilic Subtilisin-Like Proteases From Thermococcus kodakarensis. Biotechnology of Microbial Enzymes: Production, Biocatalysis and Industrial Applications.

[43] Chohan S.M., Sajed M., Naeem S.U., Rashid N. (2020). Heterologous gene expression and characterization of TK2246, a highly active and thermostable plant type l-asparaginase from Thermococcus kodakarensis. Int. J. Biol. Macromol..

[44] Kengen S.W.M. (2017). ‘*Pyrococcus furiosus*, 30 years on’. Microb. Biotechnol..

[45] Amo T., Paje M.L.F., Inagaki A., Ezaki S., Atomi H., Imanaka T. (2002). *Pyrobaculum calidifontis* sp. nov., a novel hyperthermophilic archaeon that grows in atmospheric air. Archaea.

[46] Hatanaka T., Usuki H., Arima J., Uesugi Y., Yamamoto Y., Kumagai Y., Yamasato A., Mukaihara T. (2010). Extracellular Production and Characterization of Two Streptomyces l-Asparaginases. Appl. Biochem. Biotechnol..

[47] Pritsa A.A., Kyriakidis D.A. (2001). L-asparaginase of Thermus thermophilus: Purification, properties and identificaation of essential amino acids for its catalytic activity. Mol. Cell. Biochem..

[48] Pritsa A., Choli-Papadopoulou T., Kyriakidis D.A. (1998). Studies on the primary structure of L-asparaginase from Thermus thermophilus. Protein J..

[49] Dumina M., Zhgun A., Pokrovskaya M., Aleksandrova S., Zhdanov D., Sokolov N., El’Darov M. (2021). Highly Active Thermophilic L-Asparaginase from *Melioribacter roseus* Represents a Novel Large Group of Type II Bacterial L-Asparaginases from Chlorobi-Ignavibacteriae-Bacteroidetes Clade. Int. J. Mol. Sci..

[50] Curran M.P., Daniel R.M., Guy G.R., Morgan H.W. (1985). A specific l-asparaginase from Thermus aquaticus. Arch. Biochem. Biophys..

[51] da Silva L.S., Doonan L.B., Pessoa A., de Oliveira M.A., Long P.F. (2021). Structural and functional diversity of asparaginases: Overview and recommendations for a revised nomenclature. Biotechnol. Appl. Biochem..

[52] Bentahir M., Feller G., Aittaleb M., Lamotte-Brasseur J., Himri T., Chessa J.-P., Gerday C. (2000). Structural, Kinetic, and Calorimetric Characterization of the Cold-active Phosphoglycerate Kinase from the Antarctic *Pseudomonas* sp. TACII18. J. Biol. Chem..

[53] Thomas T.M., Scopes R.K. (1998). The Effects of Temperature on the Kinetics and Stability of Mesophilic and Thermophilic 3-Phosphoglycerate Kinases. Biochem. J..

[54] Copeland W.H., Nealon D.A., Rej R. (1985). Effects of temperature on measurement of alkaline phosphatase activity. Clin. Chem..

[55] Abubakar M., Wasagu R., Umar M. (2013). Kinetic Studies of Alkaline Phosphatase from the Liver of Agama agama Lizard. Niger. J. Basic Appl. Sci..

[56] Mahesh M., Neha G., Rajesh T.S., Somashekhar R., Puttaiah E.T. (2010). Isolation and characterization of extracellular thermostable alkaline phosphatase enzyme from *Bacillus* spp.. Int. J. Appl. Biol. Pharm. Technol..

[57] Singh A.K., Pindi P.K., Dube S., Sundareswaran V.R., Shivaji S. (2009). Importance of *trmE* for Growth of the Psychrophile *Pseudomonas syringae* at Low Temperatures. Appl. Environ. Microbiol..

[58] Richer H.B., Brewer J., Fahlman G.G., Gibson B., Hansen B.M., Ibata R., Kalirai J.S., Limongi M., Rich R.M., Saviane I. (2002). The Lower Main Sequence and Mass Function of the Globular Cluster Messier 4. Astrophys. J..

[59] Li X., Zhang X., Xu S., Xu M., Yang T., Wang L., Zhang H., Fang H., Osire T., Rao Z. (2019). Insight into the thermostability of thermophilic L-asparaginase and non-thermophilic L-asparaginase II through bioinformatics and structural analysis. Appl. Microbiol. Biotechnol..

[60] Lei S., Rossi S., Huang B. (2022). Metabolic and Physiological Regulation of Aspartic Acid-Mediated Enhancement of Heat Stress Tolerance in Perennial Ryegrass. Plants.

[61] Mesas J.M., Gil J.A., Martin J.F. (1990). Characterization and Partial Purification of L-Asparaginase from Corynebacterium Glutamicum. J. Gen. Microbiol..

[62] Batool T., Makky E.A., Jalal M., Yusoff M.M. (2016). A Comprehensive Review on l-Asparaginase and Its Applications. Appl. Biochem. Biotechnol..

[63] Azevedo R.A., Arruda P., Turner W.L., Lea P.J. (1997). The biosynthesis and metabolism of the aspartate derived amino acids in higher plants. Phytochemistry.

[64] Isogai S., Takagi H. (2021). Enhancement of lysine biosynthesis confers high-temperature stress tolerance to *Escherichia coli* cells. Appl. Microbiol. Biotechnol..

[65] Kishor P.B.K., Suravajhala R., Rajasheker G., Marka N., Shridhar K.K., Dhulala D., Scinthia K.P., Divya K., Doma M., Edupuganti S. (2020). Lysine, Lysine-Rich, Serine, and Serine-Rich Proteins: Link Between Metabolism, Development, and Abiotic Stress Tolerance and the Role of ncRNAs in Their Regulation. Front. Plant Sci..

[66] Sullivan L.B., Gui D.Y., Hosios A.M., Bush L.N., Freinkman E., Vander Heiden M.G. (2015). Supporting Aspartate Biosynthesis Is an Essential Function of Respiration in Proliferating Cells. Cell.

[67] Schubert C., Zedler S., Strecker A., Unden G. (2021). L-Aspartate as a high-quality nitrogen source in *Escherichia coli*: Regulation of L-aspartase by the nitrogen regulatory system and interaction of L-aspartase with GlnB. Mol. Microbiol..

[68] Sobat M., Asad S., Kabiri M., Mehrshad M. (2021). Metagenomic discovery and functional validation of L-asparaginases with anti-leukemic effect from the Caspian Sea. Iscience.

[69] Van De Casteele M., Demarez M., Legrain C., Glansdorff N., Pierard A. (1990). Pathways of arginine biosynthesis in extreme thermophilic archaeo- and eubacteria. J. Gen. Microbiol..

[70] Yoshida A., Tomita T., Atomi H., Kuzuyama T., Nishiyama M. (2016). Lysine Biosynthesis of Thermococcus kodakarensis with the Capacity to Function as an Ornithine Biosynthetic System. J. Biol. Chem..

[71] Oshima T. (2007). Unique polyamines produced by an extreme thermophile, Thermus thermophilus. Amino Acids.

[72] Fukuda W., Morimoto N., Imanaka T., Fujiwara S. (2008). Agmatine is essential for the cell growth of *Thermococcus kodakaraensis*. FEMS Microbiol. Lett..

[73] Morimoto N., Fukuda W., Nakajima N., Masuda T., Terui Y., Kanai T., Oshima T., Imanaka T., Fujiwara S. (2010). Dual Biosynthesis Pathway for Longer-Chain Polyamines in the Hyperthermophilic Archaeon *Thermococcus kodakarensis*. J. Bacteriol..

[74] Michael A.J. (2018). Polyamine function in archaea and bacteria. J. Biol. Chem..

[75] Nakashima M., Yamagami R., Tomikawa C., Ochi Y., Moriya T., Asahara H., Fourmy D., Yoshizawa S., Oshima T., Hori H. (2017). Long and branched polyamines are required for maintenance of the ribosome, tRNA^His^ and tRNA^Tyr^ in *Thermus thermophilus* cells at high temperatures. Genes Cells.

[76] Nishio T., Yoshikawa Y., Fukuda W., Umezawa N., Higuchi T., Fujiwara S., Imanaka T., Yoshikawa K. (2018). Branched-Chain Polyamine Found in Hyperthermophiles Induces Unique Temperature-Dependent Structural Changes in Genome-Size DNA. ChemPhysChem.

[77] Ichikawa J.K., Clarke S. (1998). A Highly Active Protein Repair Enzyme from an Extreme Thermophile: Thel-Isoaspartyl Methyltransferase fromThermotoga maritima. Arch. Biochem. Biophys..

[78] La J.W., Dhanasingh I., Jang H., Lee S.H., Lee D.-W. (2020). Functional Characterization of Primordial Protein Repair Enzyme M38 Metallo-Peptidase From Fervidobacterium islandicum AW-1. Front. Mol. Biosci..

[79] Si M., Xu Q., Jiang L., Huang H. (2016). SpyTag/SpyCatcher Cyclization Enhances the Thermostability of Firefly Luciferase. PLoS ONE.

[80] Jahns T. (1996). Ammonium/urea-dependent generation of a proton electrochemical potential and synthesis of ATP in Bacillus pasteurii. J. Bacteriol..

[81] Singh J., Khan M.I., Yadav S.P., Srivastava A., Sinha K.K., Das P., Kundu B. (2017). L-Asparaginase of Leishmania donovani: Metabolic target and its role in Amphotericin B resistance. Int. J. Parasitol. Drugs Drug Resist..

[82] Salbitani G., Carfagna S. (2021). Ammonium Utilization in Microalgae: A Sustainable Method for Wastewater Treatment. Sustainability.

[83] Konings W.N., Albers S.-V., Koning S., Driessen A.J.M. (2002). The cell membrane plays a crucial role in survival of bacteria and archaea in extreme environments. Int. J. Gen. Mol. Microbiol..

[84] Stetter K.O. (1999). Extremophiles and their adaptation to hot environments. FEBS Lett..

[85] Lu P., Ma D., Chen Y., Guo Y., Chen G.-Q., Deng H., Shi Y. (2013). L-glutamine provides acid resistance for Escherichia coli through enzymatic release of ammonia. Cell Res..

[86] Lamosa P., Burke A., Peist R., Huber R., Liu M.-Y., Silva G., Rodrigues-Pousada C., LeGall J., Maycock C., Santos H. (2000). Thermostabilization of Proteins by Diglycerol Phosphate, a New Compatible Solute from the Hyperthermophile *Archaeoglobus fulgidus*. Appl. Environ. Microbiol..

[87] Shi R., Liu Y., Mu Q., Jiang Z., Yang S. (2017). Biochemical characterization of a novel L-asparaginase from Paenibacillus barengoltzii being suitable for acrylamide reduction in potato chips and mooncakes. Int. J. Biol. Macromol..

[88] Jia M., Xu M., He B., Rao Z. (2013). Cloning, Expression, and Characterization of l-Asparaginase from a Newly Isolated Bacillus subtilis B11–06. J. Agric. Food Chem..

[89] Unsworth L., Van Der Oost J., Koutsopoulos S. (2007). Hyperthermophilic enzymes − stability, activity and implementation strategies for high temperature applications. FEBS J..

[90] Feng C., Ma Z., Yang D., Li X., Zhang J., Li Y. (2020). A Method for Prediction of Thermophilic Protein Based on Reduced Amino Acids and Mixed Features. Front. Bioeng. Biotechnol..

[91] Vieille C., Zeikus G.J. (2001). Hyperthermophilic Enzymes: Sources, Uses, and Molecular Mechanisms for Thermostability. Microbiol. Mol. Biol. Rev..

[92] De Farias S.T., Bonato M.C.M. (2002). Preferred codons and amino acid couples in hyperthermophiles. Genome Biol..

[93] Cambillau C., Claverie J.-M. (2000). Structural and Genomic Correlates of Hyperthermostability. J. Biol. Chem..

[94] Taylor T.J., Vaisman I.I. (2010). Discrimination of thermophilic and mesophilic proteins. BMC Struct. Biol..

[95] Kumar S., Tsai C.-J., Nussinov R. (2000). Factors enhancing protein thermostability. Protein Eng..

[96] Szilágyi A., Závodszky P. (2000). Structural differences between mesophilic, moderately thermophilic and extremely thermophilic protein subunits: Results of a comprehensive survey. Structure.

[97] Chan C.-H., Yu T.-H., Wong K.-B. (2011). Stabilizing Salt-Bridge Enhances Protein Thermostability by Reducing the Heat Capacity Change of Unfolding. PLoS ONE.

[98] Long S., Zhang X., Rao Z., Chen K., Xu M., Yang T., Yang S. (2016). Amino acid residues adjacent to the catalytic cavity of tetramer l-asparaginase II contribute significantly to its catalytic efficiency and thermostability. Enzym. Microb. Technol..

[99] Leng F., Wu L.-Y., Lu C., Pan X.-M. (2017). Determinants of Thermostability in Serine Hydroxymethyltransferase Identified by Principal Component Analysis. Sci. Rep..

[100] Bhatt A.N., Khan M.Y., Bhakuni V. (2004). The C-terminal domain of dimeric serine hydroxymethyltransferase plays a key role in stabilization of the quaternary structure and cooperative unfolding of protein: Domain swapping studies with enzymes having high sequence identity. Protein Sci..

[101] Sharma P., Tomar R., Yadav S.S., Badmalia M.D., Nath S.K., Kundu B. (2020). Heat induces end to end repetitive association in P. furiosus l-asparaginase which enables its thermophilic property. Sci. Rep..

[102] Tomar R., Sharma P., Srivastava A., Bansal S., Kundu B. (2014). Structural and functional insights into an archaealL-asparaginase obtained through the linker-less assembly of constituent domains. Acta Crystallogr. Sect. D Biol. Crystallogr..

[103] Álvarez-Cao M.-E., González R., Pernas M.A., Rúa M.L. (2018). Contribution of the Oligomeric State to the Thermostability of Isoenzyme 3 from Candida rugosa. Microorganisms.

[104] Reed C.J., Lewis H., Trejo E., Winston V., Evilia C. (2013). Protein Adaptations in Archaeal Extremophiles. Archaea.

[105] Anand S., Sharma C. (2018). Glycine-rich loop encompassing active site at interface of hexameric M. tuberculosis Eis protein contributes to its structural stability and activity. Int. J. Biol. Macromol..

[106] Atkinson S.C., Dogovski C., Wood K., Griffin M.D., Gorman M.A., Hor L., Reboul C.F., Buckle A.M., Wuttke J., Parker M.W. (2018). Substrate Locking Promotes Dimer-Dimer Docking of an Enzyme Antibiotic Target. Structure.

[107] Welin M., Lehtiö L., Johansson A., Flodin S., Nyman T., Trésaugues L., Hammarström M., Gräslund S., Nordlund P. (2013). Substrate Specificity and Oligomerization of Human GMP Synthetase. J. Mol. Biol..

[108] Aprile F.A., Dhulesia A., Stengel F., Roodveldt C., Benesch J.L.P., Tortora P., Robinson C.V., Salvatella X., Dobson C.M., Cremades N. (2013). Hsp70 Oligomerization Is Mediated by an Interaction between the Interdomain Linker and the Substrate-Binding Domain. PLoS ONE.

[109] Qiu L., Gulotta M., Callender R. (2007). Lactate Dehydrogenase Undergoes a Substantial Structural Change to Bind its Substrate. Biophys. J..

