# Supplementary material for: Thermo-L-Asparaginases: From the Role in the Viability of Thermophiles and Hyperthermophiles at High Temperatures to a Molecular Understanding of Their Thermoactivity and Thermostability
Source: Int J Mol Sci. 2023 Jan 31;24(3):2674. doi: 10.3390/ijms24032674 (PMC9916696; doi:10.3390/ijms24032674)
Supplement: Supplementary file 1 [file ijms-24-02674-s001.zip › ijms-2070922-supplementary.pdf]

Supplementary Materials

Table S1. Relative amino acid compositions of mesophilic and Thermo-ASNases

**Residue(s)**	**Proteins**		**L-ASNase**												
Residue(s)	MP [91]	TP/HP [91]	**EcAI**	*TkAI*	*TsAI*	*TzAI*	*PhAI*	*PfAI*	*PyAI*	**EcAII**	*MrAII*	*StAII*	**EcAIII**	*TkAIII*	*MrAIII*
Ala	8.09 ± 1.54	6.82 ± 1.42	**8.58**	*8.84*	*6.65*	*8.49*	*6.10*	*6.14*	*7.01*	**10.92**	*5.83*	*17.75*	**14.64**	*12.42*	*10.56*
Cys	1.10 ± 0.18	0.86 ± 0.27	**0.30**	*0.00*	*0.30*	*0.00*	*0.00*	*0.61*	*0.00*	**0.58**	*0.92*	*1.18*	**1.56**	*0.33*	*0.62*
Asp	5.06 ± 0.18	4.63 ± 0.54	**5.33**	*5.79*	*5.74*	*5.46*	*5.18*	*6.44*	*4.88*	**7.76**	*5.52*	*5.62*	**4.36**	*4.90*	*5.59*
Glu	6.45 ± 0.54	8.55 ± 0.95	**5.33**	*6.71*	*6.34*	*7.27*	*7.01*	*6.44*	*8.23*	**2.01**	*8.59*	*5.62*	**7.79**	*9.48*	*5.28*
Phe	4.61 ± 0.78	4.40 ± 0.82	**2.96**	*2.44*	*2.1*	*3.03*	*3.05*	*2.76*	*2.13*	**3.16**	*4.60*	*1.78*	**2.18**	*3.27*	*5.59*
Gly	6.70 ± 0.96	7.16 ± 0.68	**8.28**	*9.76*	*8.16*	*10.91*	*9.45*	*9.20*	*9.76*	**8.62**	*8.59*	*9.76*	**12.15**	*12.09*	*11.80*
His	2.04 ± 0.21	1.57 ± 0.16	**3.55**	*1.52*	*1.21*	*2.12*	*0.92*	*0.92*	*1.22*	**0.86**	*1.53*	*2.96*	**1.87**	*1.31*	*1.55*
Ile	7.40 ± 1.69	7.82 ± 1.64	**6.51**	*7.62*	*11.18*	*6.97*	*11.28*	*9.20*	*5.79*	**3.74**	*9.51*	*2.37*	**4.05**	*6.21*	*7.45*
Lys	6.81 ± 2.00	7.61 ± 2.16	**2.96**	*3.96*	*10.27*	*6.06*	*7.01*	*6.14*	*3.66*	**6.90**	*7.98*	*1.48*	**2.80**	*8.50*	*7.45*
Leu	10.43 ± 0.55	10.21 ± 0.68	**10.06**	*10.37*	*8.46*	*9.70*	*9.76*	*10.12*	*11.59*	**7.47**	*7.98*	*11.24*	**9.66**	*7.52*	*8.70*
Met	2.42 ± 0.28	2.29 ± 0.25	**3.55**	*3.05*	*4.23*	*2.73*	*3.66*	*3.68*	*3.96*	**2.30**	*2.76*	*2.07*	**4.05**	*3.60*	*3.73*
Asn	4.90 ± 1.20	3.52 ± 0.94	**5.03**	*3.05*	*4.23*	*2.42*	*2.74*	*4.29*	*2.44*	**6.61**	*6.44*	*1.18*	**3.12**	*2.29*	*6.21*
Pro	3.77 ± 0.77	4.36 ± 0.99	**6.51**	*5.49*	*4.53*	*5.46*	*3.96*	*3.99*	*5.49*	**3.74**	*4.29*	*7.10*	**3.12**	*2.61*	*2.17*
Gln	3.99 ± 0.75	1.78 ± 0.22	**4.73**	*1.22*	*0.91*	*1.21*	*1.22*	*1.23*	*0.92*	**3.74**	*0.92*	*2.37*	**2.49**	*0.65*	*1.86*

Table S1. Cont.

**Residue(s)**	**Proteins**		**L-ASNase**												
Residue(s)	MP [91]	TP/HP [91]	**EcAI**	*TkAI*	*TsAI*	*TzAI*	*PhAI*	*PfAI*	*PyAI*	**EcAII**	*MrAII*	*StAII*	**EcAIII**	*TkAIII*	*MrAIII*
Arg	4.33 ± 0.98	5.57 ± 1.16	**4.44**	*7.01*	*3.02*	*4.24*	*5.18*	*4.60*	*6.71*	**2.30**	*2.15*	*7.99*	**4.67**	*4.25*	*3.73*
Ser	6.08 ± 0.57	5.54 ± 1.01	**4.73**	*4.27*	*4.23*	*5.46*	*6.71*	*4.91*	*3.66*	**4.89**	*5.22*	*4.73*	**4.98**	*3.27*	*4.66*
Thr	5.09 ± 0.57	4.34 ± 0.23	**6.81**	*7.93*	*7.55*	*6.67*	*5.18*	*6.75*	*6.40*	**10.06**	*6.14*	*2.96*	**5.92**	*5.56*	*3.73*
Val	6.35 ± 0.75	8.05 ± 0.68	**5.92**	*7.32*	*6.04*	*8.18*	*7.01*	*8.59*	*11.59*	**10.63**	*7.06*	*10.06*	**7.79**	*8.82*	*6.21*
Trp	1.02 ± 0.31	1.06 ± 0.20	**0.30**	*0.61*	*0.60*	*0.61*	*0.92*	*0.61*	*1.52*	**0.29**	*0.61*	*0.89*	**0.31**	*1.31*	*0.00*
Tyr	3.30 ± 0.43	3.82 ± 0.33	**4.14**	*3.05*	*4.23*	*3.03*	*3.66*	*3.37*	*3.05*	**3.45**	*3.37*	*0.89*	**2.49**	*1.63*	*3.11*
Charged residues (DEKRH)	24.69	27.93	**21.60**	*25.00*	*26.59*	*25.15*	*25.31*	*24.54*	*24.70*	**19.83**	*25.77*	*23.67*	**21.50**	*28.43*	*23.60*
Polar/uncharged residues (GSTNQYC)	31.26	27.02	**34.02**	*29.28*	*29.61*	*29.33*	*28.96*	*30.36*	*23.18*	**37.94**	*31.59*	*23.08*	**32.71**	*25.81*	*31.99*
Hydrophobic residues (LMIVWPAF)	44.09	45.01	**44.39**	*45.74*	*43.81*	*45.16*	*45.74*	*45.12*	*49.08*	**42.25**	*42.63*	*53.26*	**45.80**	*45.76*	*44.41*
Aromatic residues (F+H+W+Y)	10.97	10.85	**10.95**	*7.63*	*8.16*	*8.79*	*8.54*	*7.67*	*7.93*	**7.76**	*10.12*	*6.51*	**6.85**	*7.52*	*10.25*

mesophilic L-ASNases (EcAI, EcAII, EcAIII) are in bold, thermo-ASNases are in italics. Decreased and increased amino acid content in thermo-ASNases compared to mesophilic counterparts is marked in red and green, respectively.
